# Acetylation-induced degradation of ECHS1 enhances BCAA accumulation and proliferation in KRAS-mutant colorectal cancer

**DOI:** 10.1186/s13046-025-03399-3

**Published:** 2025-05-28

**Authors:** Zhenkang Li, Zhengyu Liu, Mingdao Lin, Huayang Pan, Yuechen Liu, Yang Liu, Yuwen Xie, Jinchao Zhang, Shenyuan Guan, Yongsheng Li, Mulan Zhu, Yuan Fang, Zhiyong Shen, Haijun Deng

**Affiliations:** 1 Department of General Surgery, Nanfang Hospital, Southern Medical University, Guangzhou, Guangdong Province 510515 China; 2 Department of Anorectal Surgery, Hainan General Hospital, Affiliated Hainan Hospital of Hainan Medical University, Haikou, Hainan Province 570311 China; 3 Department of Radiation Oncology, Hainan General Hospital, Affiliated Hainan Hospital of Hainan Medical University, Haikou, Hainan Province 570311 China; 4 Department of Radiation Oncology, Nanfang Hospital, Southern Medical University, Guangzhou, Guangdong Province 510515 China

**Keywords:** Colorectal cancer, KRAS, BCAAs, Acetylation

## Abstract

**Background:**

Branched-chain amino acid (BCAA) metabolism is dysregulated in colorectal cancer (CRC), with elevated plasma BCAA levels significantly associated with an increased risk of developing the disease. However, whether BCAAs directly promote CRC progression and their underlying mechanisms remain unclear.

**Methods:**

In this study, we investigated the metabolic alterations in KRAS-mutant CRC. We examined the effects of restricting BCAA supply on the proliferation and metastasis of KRAS-mutant CRC cells both in vitro and in vivo.

**Results:**

We found that in KRAS-mutant CRC, BCAAs and their metabolic products accumulate markedly. Restricting the BCAA supply specifically inhibits the proliferation of KRAS-mutant CRC cells but does not affect metastasis. In these cancer cells, enoyl-CoA hydratase-1 (ECHS1), a key enzyme in BCAA metabolism, is downregulated. Furthermore, BCAAs enhance the acetylation of lysine 204 on ECHS1, impairing its ability to bind enoyl-CoA and reducing its catalytic activity. This modification triggers the ubiquitination of ECHS1 and its subsequent degradation, diminishing BCAA catabolism and leading to its cellular accumulation. This accumulation activates the mTORC1 signaling pathway, which induces the transcriptional activation of downstream target proteins and promotes the malignant progression of CRC.

**Conclusions:**

Limiting BCAA intake not only suppresses tumor growth in KRAS-mutant CRC but also enhances the efficacy of the KRAS G12D inhibitor MRTX1133 and the monoclonal antibody bevacizumab. Our findings reveal a previously unknown regulatory mechanism of ECHS1 in CRC and offer new potential therapeutic targets.

**Supplementary Information:**

The online version contains supplementary material available at 10.1186/s13046-025-03399-3.

## Introduction

Colorectal cancer (CRC) is one of the most prevalent malignancies worldwide and is associated with high incidence and mortality rates [1]. A major challenge in treating CRC is the high prevalence of KRAS mutations, which occur in approximately 40% of patients [2]. These mutations are closely linked to poor prognosis and resistance to standard treatments such as epidermal growth factor receptor (EGFR) inhibitors [3]. KRAS mutations promote tumor cell proliferation and survival by activating key signaling pathways, including the PI3K/Akt and MAPK pathways, and modulating the cellular response to chemotherapy, often by reducing drug-induced apoptosis or altering drug metabolism [4, 5].

Recent research has emphasized the critical role of KRAS in driving metabolic reprogramming in several cancers, including pancreatic, colorectal, and lung cancers. KRAS mutations activate glycolysis and redirect intermediates to key biosynthetic pathways, including the pentose phosphate and serine synthesis pathways, which supply nucleotides and reducing equivalents for cell proliferation [6–8]. Additionally, KRAS mutations enhance amino acid metabolism, especially glutamine metabolism, to sustain redox balance within tumor cells [9–12]. Furthermore, these mutations regulate fatty acid β-oxidation and de novo synthesis, contributing to malignant proliferation and resistance to cell death by altering lipid metabolism [13].

Branched-chain amino acids (BCAAs), including leucine, isoleucine, and valine, are essential amino acids that cannot be synthesized by the human body but can be metabolized through a series of highly reversible enzymatic reactions [14]. The metabolism of BCAAs is notably altered in various solid tumors, including melanoma, nasopharyngeal carcinoma, breast cancer, and liver cancer [15–18]. Pancreatic cancers with KRAS mutations show dysregulated BCAA catabolism, leading to the accumulation of BCAA-derived acetyl-CoA, which significantly affects disease onset and progression [19]. Reducing dietary BCAA levels significantly suppressed pancreatic cancer development in mouse models with KRAS mutations [20, 21]. However, the precise roles and mechanisms of BCAAs in KRAS-mutated CRC remain unclear.

Lysine acetylation, a key posttranslational modification, is controlled by the reversible activity of histone acetyltransferases (HATs) and histone deacetylases (HDACs). This modification plays a vital role in regulating various cellular processes, including gene transcription, the cell cycle, and signaling pathways [22]. Lysine acetylation in mitochondria is essential for controlling metabolic processes by regulating the activity of metabolic enzymes, thereby affecting carbon source utilization and metabolic pathway flux [23]. Enoyl-CoA hydratase short-chain 1 (ECHS1), which links BCAA metabolism to fatty acid pathways, can sense nutrients and increase tumor resistance to apoptosis by promoting its acetylation [24].

In this study, we explored the impact of BCAAs on the proliferation and molecular pathways of KRAS-mutant CRC. Our findings elucidate the pivotal roles of BCAAs and ECHS1 in modulating tumor growth and highlight potential targets for therapeutic intervention.

## Results

### BCAAs are significantly enriched in KRAS-mutated CRC

**Fig. 1:**
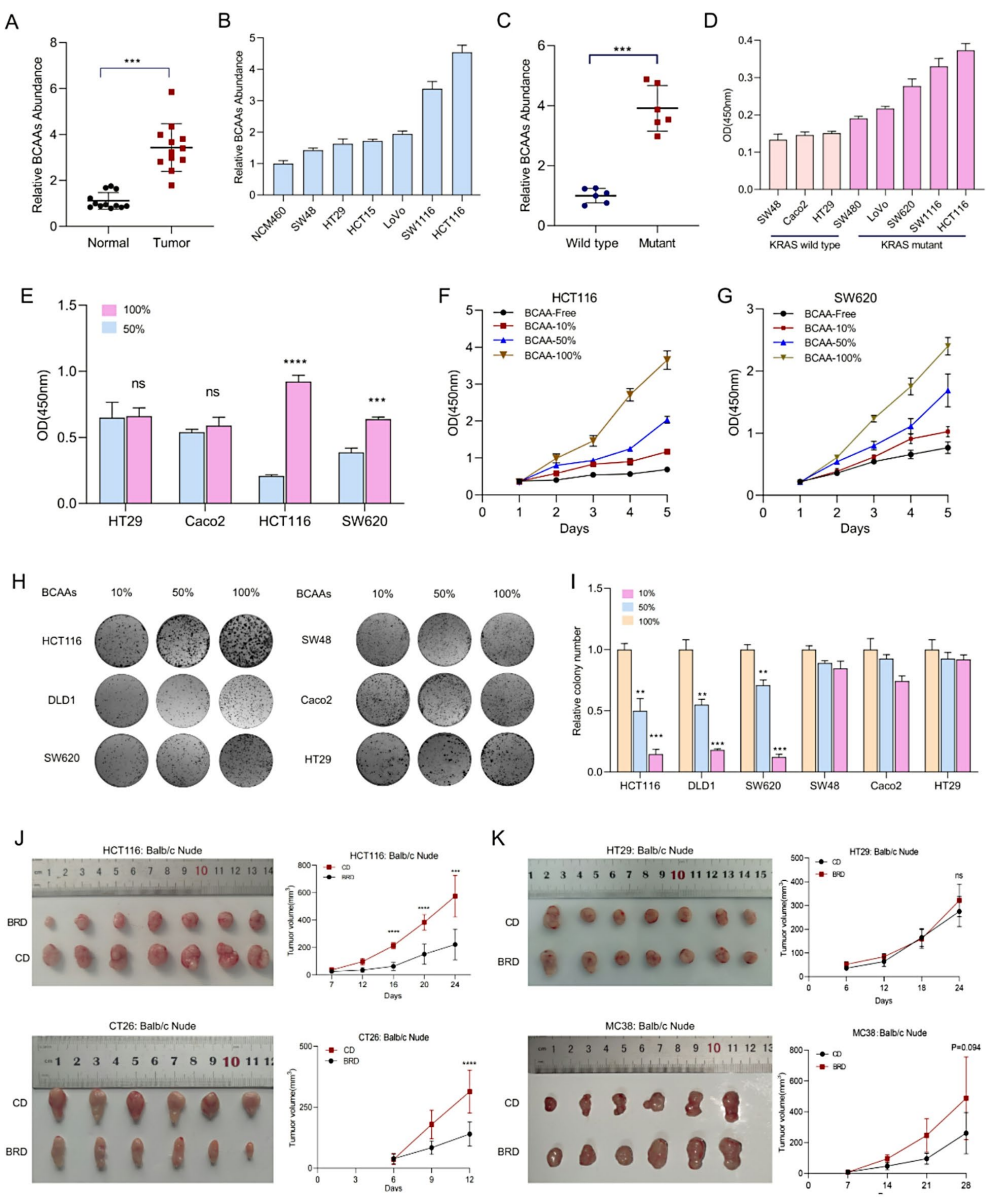
BCAA enrichment promotes the progression of KRAS-mutant CRC. **A** Quantification of BCAA levels in twelve pairs of CRC tissues and corresponding normal colonic epithelial tissues using a BCAA kit. **B** Comparison of BCAA enrichment in CRC cell lines and normal cell lines. **C**, **D** BCAA content in KRAS wild-type and KRAS mutant CRC cells measured using a BCAA kit. **E-G** Effects of reducing or eliminating BCAAs on the proliferation rates of KRAS mutant HCT116 and SW620 cells compared with those of KRAS wild-type Caco2 and HT29 cells, assessed via CK8 assay. **H**, **I** Plate colony formation assay results demonstrating the impact of BCAA restriction on cell proliferation. **J**, **K** Effects of BRD on subcutaneous tumor proliferation in the KRAS-mutant HCT116 and CT26 models compared with the KRAS wild-type HT29 and MC38 models. Statistical analysis of subcutaneous tumor volume. BRD: BCAA-restricted diet, CD: Control diet, BCAA: Branched-chain amino acids, KRAS: Kirsten rat sarcoma viral oncogene homolog, CCK8 assay: Cell counting Kit-8 assay, DMEM: Dulbecco’s Modified Eagle Medium

We initially quantified BCAA levels in 12 pairs of tumor tissues and corresponding normal colonic epithelial tissues and observed significantly elevated BCAA levels in CRC tissues compared with their normal counterparts (Fig. 1A; Supplementary Table 1). Further cell-based assays revealed that, compared with normal cell lines, CRC cell lines presented greater BCAA enrichment (Fig. 1B). Previous research has indicated that CRC cells accumulate significant amounts of BCAAs following a KRAS mutation due to the activation of specific metabolic pathways [25]. We then compared the BCAA content between KRAS wild-type and mutant CRC cells and detected significant BCAA enrichment in KRAS mutant cells (Fig. 1C, D). These findings support the observed distinct metabolic adaptations in BCAA processing in CRC [26].

### Restricting BCAA supply inhibits the proliferation of KRAS-Mutant CRC cells without affecting metastasis

As BCAAs are essential amino acids that cannot be synthesized endogenously, we explored the effects of BCAA restriction on CRC cell proliferation in vitro. Reducing or eliminating BCAAs significantly inhibited the proliferation of the KRAS mutant HCT116 and SW620 cells but had little effect on the proliferation of the KRAS wild-type Caco2 and HT29 cells (Fig. 1E–G). The plate colony formation assays confirmed these findings (Fig. 1H, I). We also investigated whether dietary BCAA restriction could suppress CRC proliferation in vivo. A BCAA-restricted diet (BRD) significantly reduced subcutaneous tumor growth in the KRAS-mutant HCT116 and CT26 models, with no notable effect on KRAS wild-type HT29 and MC38 tumors (Fig. 1J, K). Next, we examined the effect of BCAA restriction on the metastasis of KRAS-mutant CRC cells. Scratch assays and transwell migration experiments revealed that limiting BCAAs did not significantly affect the migration of HCT116 and SW620 cells (Supplementary Fig. 1A, B). Similarly, spleen-to-liver metastasis assays revealed that dietary BCAA restriction did not influence metastatic behavior of CRC cells (Supplementary Fig. 1C, D). These results indicate that restricting BCAA supply inhibits the proliferation of KRAS-mutant CRC cells without affecting metastasis.

### ECHS1 is a key gene in BCAA metabolism and is downregulated in KRAS-mutant CRC

**Fig. 2:**
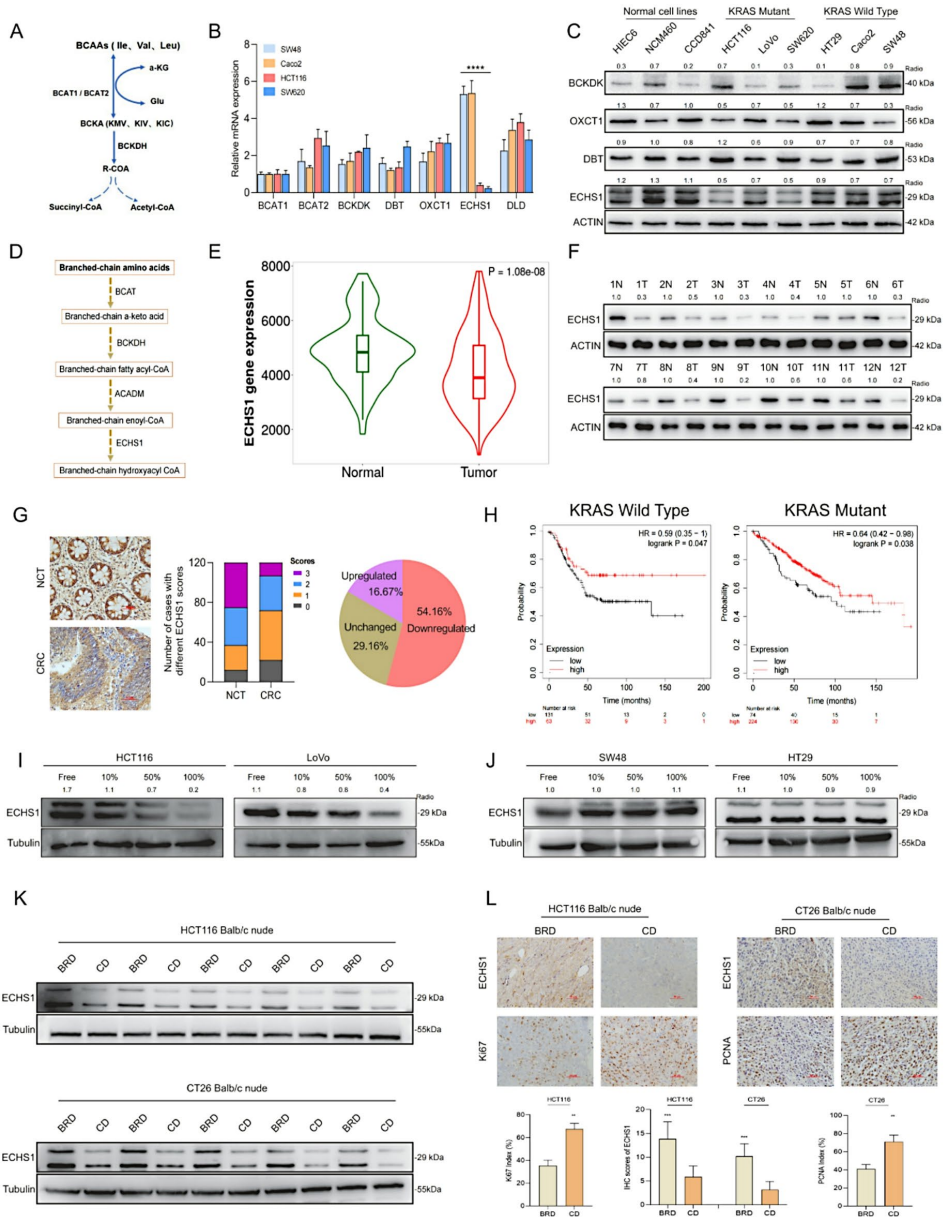
Function and regulation of ECHS1 in KRAS-mutant CRC. **A**, **B** mRNA expression levels of ECHS1 in KRAS mutant HCT116 and SW620 cells compared with those in KRAS wild-type Caco2 and SW48 cells. **C** Western blot analysis of ECHS1 protein expression in KRAS mutant versus wild-type CRC cells and normal cells. **D** The metabolic pathway of BCAAs and associated key enzymes and metabolites. **E** mRNA expression levels of ECHS1 in CRC samples from the TNM plot dataset. **F** Western blot analysis of ECHS1 protein levels in 12 paired CRC tissue samples. **G** IHC staining showing ECHS1 expression in 120 paired CRC tissues. **H** Correlation between ECHS1 expression levels and overall survival rates in CRC patients with KRAS wild-type and KRAS-mutant genotypes. **I**, **J** Western blot analysis of ECHS1 protein expression in HCT116 and LoVo cells treated with various BCAA concentrations compared with that in SW48 and HT29 cells. **K**, **L** Effects of different BCAA diets on ECHS1 expression levels and proliferation markers (Ki67 and PCNA) in subcutaneous tumors in mice. ECHS1: Enoyl-CoA hydratase-1, KRAS: Kirsten rat sarcoma viral oncogene homolog, CRC: Colorectal cancer, mRNA: Messenger RNA, BCAA: Branched-chain amino acids, Ki67: Ki67 antigen, PCNA: Proliferating cell nuclear antigen, TNM: Tumor, Node, Metastasis Classification of Malignant Tumors, IHC: Immunohistochemistry

To explore the variability in BCAA metabolism associated with CRC, we analyzed the mRNA expression of key enzymes involved in BCAA transport and metabolism. We detected significantly lower ECHS1 transcription levels in KRAS mutant HCT116 and SW620 cells than in KRAS wild-type Caco2 and SW48 cells (Fig. 2A, B; Supplementary Fig. 2A, B). Moreover, ECHS1 protein expression was markedly lower in KRAS-mutant CRC cells compared to KRAS wild-type and normal colon cells, supporting its downregulation in KRAS-mutant CRC (Fig. 2C). To determine the clinical relevance of ECHS1, we assessed its mRNA expression in the TNM plot dataset, revealing its downregulation in CRC samples (Fig. 2D, E). We confirmed this finding by measuring ECHS1 protein levels in 12 paired CRC tissue samples via western blotting and observed decreased ECHS1 expression in 10 cases (Fig. 2F). Furthermore, we assessed ECHS1 protein expression in 120 paired CRC tissues via immunohistochemistry and detected elevated levels in 54.16% of the samples (65/120) compared with matched noncancerous tissues (Fig. 2G). Notably, reduced ECHS1 expression levels were associated with poorer overall survival rates, independent of KRAS status. (Fig. 2H). These findings emphasize the potential role of ECHS1 in the metabolic alterations of KRAS-mutant CRC and its prognostic significance.

### Negative regulation of ECHS1 protein expression by BCAAs in KRAS-Mutant CRC

Previous reports have indicated that nutrient levels can influence ECHS1 protein expression [27]. We examined the effects of different BCAA concentrations on ECHS1 expression in HCT116 and LoVo cells via western blotting and observed decreased expression with increasing BCAA levels. In contrast, ECHS1 expression in SW48 and HT29 cells remained unchanged (Fig. 2I, J). This differential response indicated that BCAAs may specifically downregulate ECHS1 in KRAS-mutant CRC cells. We then investigated how different BCAA diets affect ECHS1 expression in subcutaneous mouse tumors. We observed that ECHS1 levels were lower in the control diet (CD) group than in the BRD group (Fig. 2K), which corresponded with an increase in the expression of the proliferation markers Ki67 and PCNA (Fig. 2L). These findings further substantiate the hypothesis that BCAAs negatively affect ECHS1 expression in KRAS-mutant CRC.

To explore ECHS1’s biological role, we modulated its expression or knocked down KRAS in HCT116 and LoVo cells using lentiviral transduction (Supplementary Fig. 2C, D). CCK-8 and EdU assays showed that KRAS knockdown abolished BCAA concentration-dependent proliferation effects (Supplementary Fig. 2E), while ECHS1 overexpression failed to significantly alter proliferation under high BCAA levels (Supplementary Fig. 2F). These results suggest that low ECHS1 expression is critical for BCAA-driven proliferation in KRAS-mutant CRC cells.

### Downregulation of ECHS1 activates PI3K-AKT-mTORC signaling

**Fig. 3:**
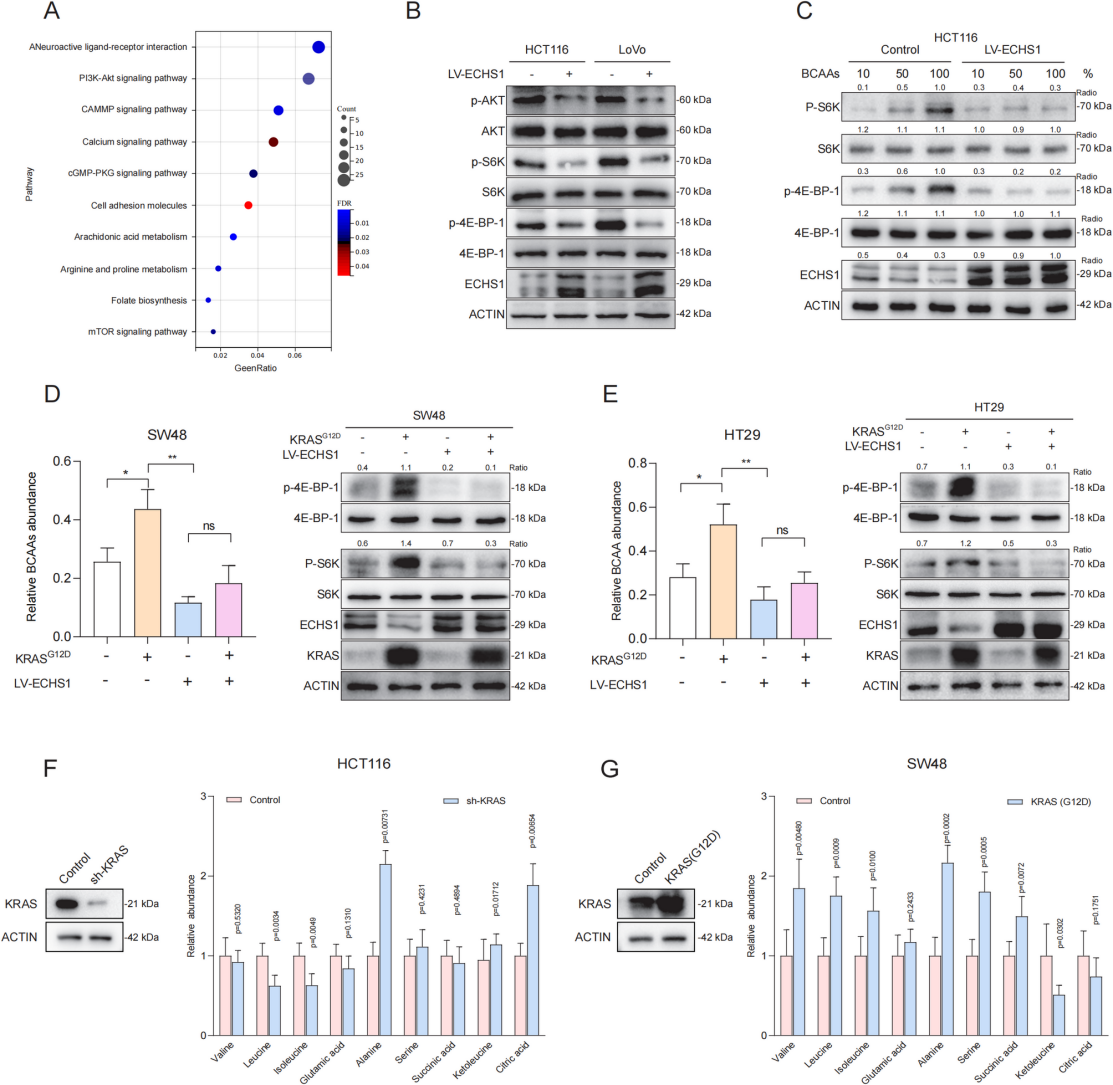
ECHS1 overexpression inhibits mTORC signaling in KRAS-mutant CRC cells. **A** KEGG enrichment analysis of significant downregulation of genes following ECHS1 overexpression in HCT116 cells. **B** Impact of ECHS1 overexpression on the expression of the mTORC downstream target proteins P-S6K and P-4EBP1 in HCT116 and LoVo cells. **C** Effects of increased BCAA concentrations on the expression of mTORC pathway downstream target proteins in HCT116 cells with low ECHS1 expression and ECHS1 overexpression. **D**, **E** Analysis of mTORC pathway activity and BCAA content in SW48 and HT29 cells with the KRAS G12D mutation and ECHS1 overexpression. **F** Comparison of BCAA-related metabolite levels in HCT116 cells with KRAS knockdown and their wild-type counterparts. **G** Targeted metabolomics analysis of SW48 cells with the KRAS G12D mutation and the control group. ECHS1: Enoyl-CoA hydratase-1, mTORC: Mechanistic target of rapamycin complex, DEGs: Differentially expressed genes, P-S6K: Phosphorylated S6 kinase, P-4EBP1: Phosphorylated 4E-binding protein 1, BCAA: Branched-chain amino acids, KRAS: Kirsten rat sarcoma viral oncogene homolog, KEGG: Kyoto Encyclopedia of Genes and Genomes

We further examined how ECHS1 expression influences the proliferation of KRAS-mutant CRC cells via BCAAs and downstream signaling pathways. Using RNA-seq and gene expression analysis, we discovered that ECHS1 overexpression in HCT116 cells significantly downregulated multiple differentially expressed genes. KEGG enrichment analysis revealed significant downregulation of genes associated with the PI3K-AKT-mTORC and cAMP signaling pathways, indicating suppression of these pathways (Fig. 3A).

To confirm the regulatory effect of ECHS1 on the mTORC signaling pathway, we assessed the effects of ECHS1 overexpression on the downstream proteins P-S6K and P-4EBP1. The results indicated that ECHS1 overexpression significantly reduced mTORC downstream target protein levels in HCT116 and LoVo cells, suggesting that it may inhibit CRC proliferation via modulation of the mTORC pathway (Fig. 3B). Moreover, higher BCAA levels increased mTORC target protein expression in HCT116 cells with low ECHS1 expression but not in those overexpressing ECHS1 (Fig. 3C). Further studies on KRAS G12D-mutant SW48 and HT29 cell lines demonstrated elevated mTORC pathway activity, indicated by increased P-S6K and P-4EBP1 protein levels. Overexpression of ECHS1 in these cells reduced intracellular BCAA levels and markedly suppressed mTORC pathway activation (Fig. 3D, E). These results highlight ECHS1’s critical role in modulating the mTORC pathway in CRC cells, potentially inhibiting KRAS-mutant CRC cell proliferation through its overexpression.

### KRAS mediated the intracellular accumulation of BCAAs in CRC

To explore the effect of KRAS on BCAA metabolism, we used targeted metabolomics and LC‒MS to compare BCAA-related metabolite levels between KRAS-knockdown HCT116 cells and control cells. KRAS knockdown in HCT116 cells significantly decreased the relative levels of leucine and isoleucine (Fig. 3F). We then introduced the KRAS G12D mutation into wild-type SW48 cells and performed targeted metabolomics on these cells and their controls. Compared with the controls, SW48 cells harboring the KRAS G12D mutation presented significantly higher levels of BCAAs and related metabolites (Fig. 3G). These findings further validated the pivotal role of KRAS in promoting BCAA accumulation in CRC cells.

### BCAAs promote acetylation at the K204 site of ECHS1

**Fig. 4:**
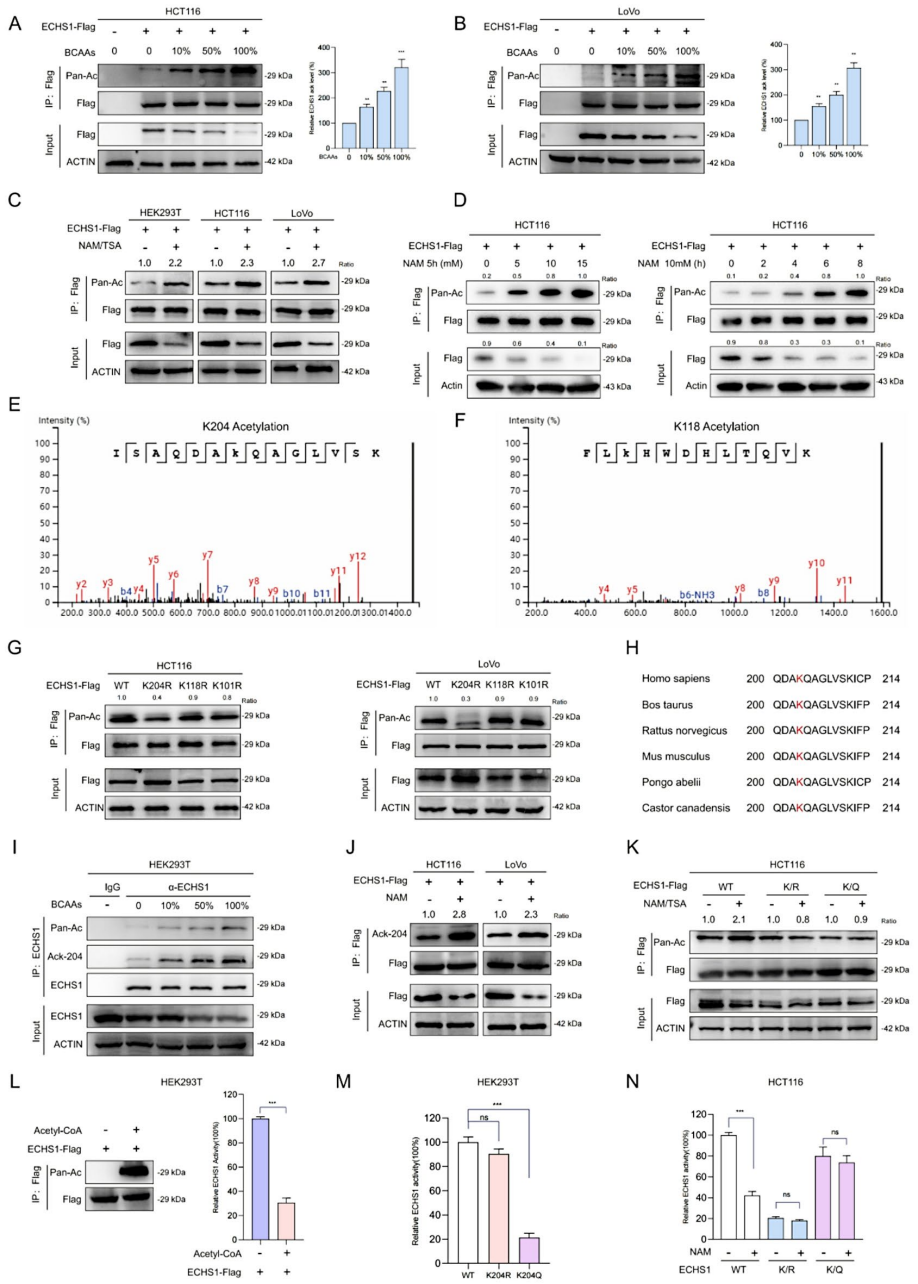
BCAAs promote the acetylation and degradation of ECHS1. **A, B** ECHS1 acetylation levels in HCT116 and LoVo cells after the addition of BCAAs. **C** Effects of the deacetylase inhibitors NAM and TSA on ECHS1 acetylation and protein levels in HEK293T, HCT116, and LoVo cells. **D** Time- and dose-dependent acetylation of ECHS1 following NAM treatment. **E**, **F** Identification of K118 and K204 as potential lysine acetylation sites on ECHS1 via mass spectrometry. **G** Effect of K204 mutation on acetylation levels of ECHS1. **H** Conservation of the K204 residue across multiple species. **I** Pan-Ac and AcK204 detection post-BCAA treatment. **J** Impact of NAM treatment on acetylation at the K204 site in HCT116 and LoVo cells. **K** Comparison of acetylation levels in wild-type ECHS1 and K204Q (K/Q) or K204R (K/R) mutants following NAM/TSA treatment. **L** Effect of acetyl-CoA on ECHS1 acetylation and catalytic activity. **M**, **N** Comparison of catalytic activity in wild-type ECHS1 and K204Q or K204R mutants following NAM treatment. ECHS1: Enoyl-CoA hydratase-1, BCAA: Branched-chain amino acids, NAM: Nicotinamide, TSA: Trichostatin A, K118: Lysine 118, K204: Lysine 204, Pan-Ac: pan-Acetylated lysine, AcK204: Acetylated lysine at position 204, K204Q: K204 glutamine mutant, K204R: K204 arginine mutant, acetyl-CoA: Acetyl coenzyme A

To investigate ECHS1 protein expression, we cultured wild-type and KRAS G12D-mutant SW48 cells in media either supplemented with BCAAs or depleted of BCAAs. Western blot analysis showed that ECHS1 expression was reduced in G12D-mutant cells under BCAA-depleted conditions and was further decreased in BCAA-supplemented media compared to BCAA-depleted media (Supplementary Fig. 3A). These observations suggest that, beyond the influence of KRAS, additional nutritional mechanisms regulate ECHS1 protein expression. Previous mass spectrometry data have identified ECHS1 as an acetylated protein capable of sensing nutrient signals [28, 29]. To explore this further, we used an anti-acetyl lysine antibody and found that BCAA supplementation increased ECHS1 acetylation in HCT116 and LoVo cells (Fig. 4A, B). Additionally, treatment with the deacetylase inhibitors nicotinamide (NAM) and trichostatin A (TSA) reduced ECHS1 protein levels while enhancing its acetylation in HEK293T, HCT116, and LoVo cells (Fig. 4C). Notably, NAM promoted ECHS1 acetylation in a time- and dose-dependent manner with pronounced effects, whereas TSA exhibited milder effects under similar conditions (Fig. 4D and Supplementary Fig. 3B, C). These findings indicate that ECHS1 acetylation, modulated by nutrient availability and deacetylase activity, may contribute to its regulation beyond KRAS-mediated pathways. HEK293T cells were chosen for their high transfection efficiency and neutral genetic background, consistent with established protocols for acetylation site identification, with findings validated in CRC cells [24, 28]. To identify the acetylation sites on the ECHS1 protein, we extracted and purified acetylated ECHS1 from HEK293T cells and identified K118 and K204 as potential lysine acetylation sites via mass spectrometry (Fig. 4E, F). Given the previous identification of K101 as a major acetylation site, we performed arginine mutation experiments at this and other sites [24]. Mutation of the K204 site significantly decreased ECHS1 acetylation levels (Fig. 4G). Notably, K204 is a conserved residue among various species (Fig. 4H). Therefore, we developed an antibody specific to acetylated ECHS1 at the K204 site. Using both a pan-acetyl lysine antibody and our specific anti-acetyl lysine 204 antibody, we observed that BCAAs increased Pan-Ac and AcK204 levels (Fig. 4I). After NAM treatment, acetylation at K204 was significantly elevated in HCT116 and LoVo cells (Fig. 4J). NAM treatment increased acetylation levels in wild-type ECHS1 but had minimal effects on the K204Q or K204R mutants, indicating a reduced acetylation signal (Fig. 4K). Thus, the K204 site was confirmed as a major acetylation site in ECHS1. 

Previous research has suggested that acetylation regulates metabolic enzyme activity and serves as a control switch [30, 31]. Therefore, we explored the specific impact of acetylation on the enzymatic activity of ECHS1. Treatment with acetyl-CoA increased intracellular ECHS1 acetylation and impaired its catalytic activity (Fig. 4L). Substituting lysine with glutamine (K/Q) at K204, which removes the positive charge, leads to a loss of ECHS1 catalytic activity. In contrast, substituting lysine with arginine (K/R), which retains the charge, had a minimal impact (Fig. 4M). In HCT116 and LoVo cells, NAM inhibited only the activity of overexpressed wild-type ECHS1, with no effect on the K/Q or K/R mutants (Fig. 4N; Supplementary Fig. 3D). These results confirmed that acetylation at K204 leads to ECHS1 inactivation.

### Acetylation-induced ubiquitination and degradation of ECHS1

**Fig. 5:**
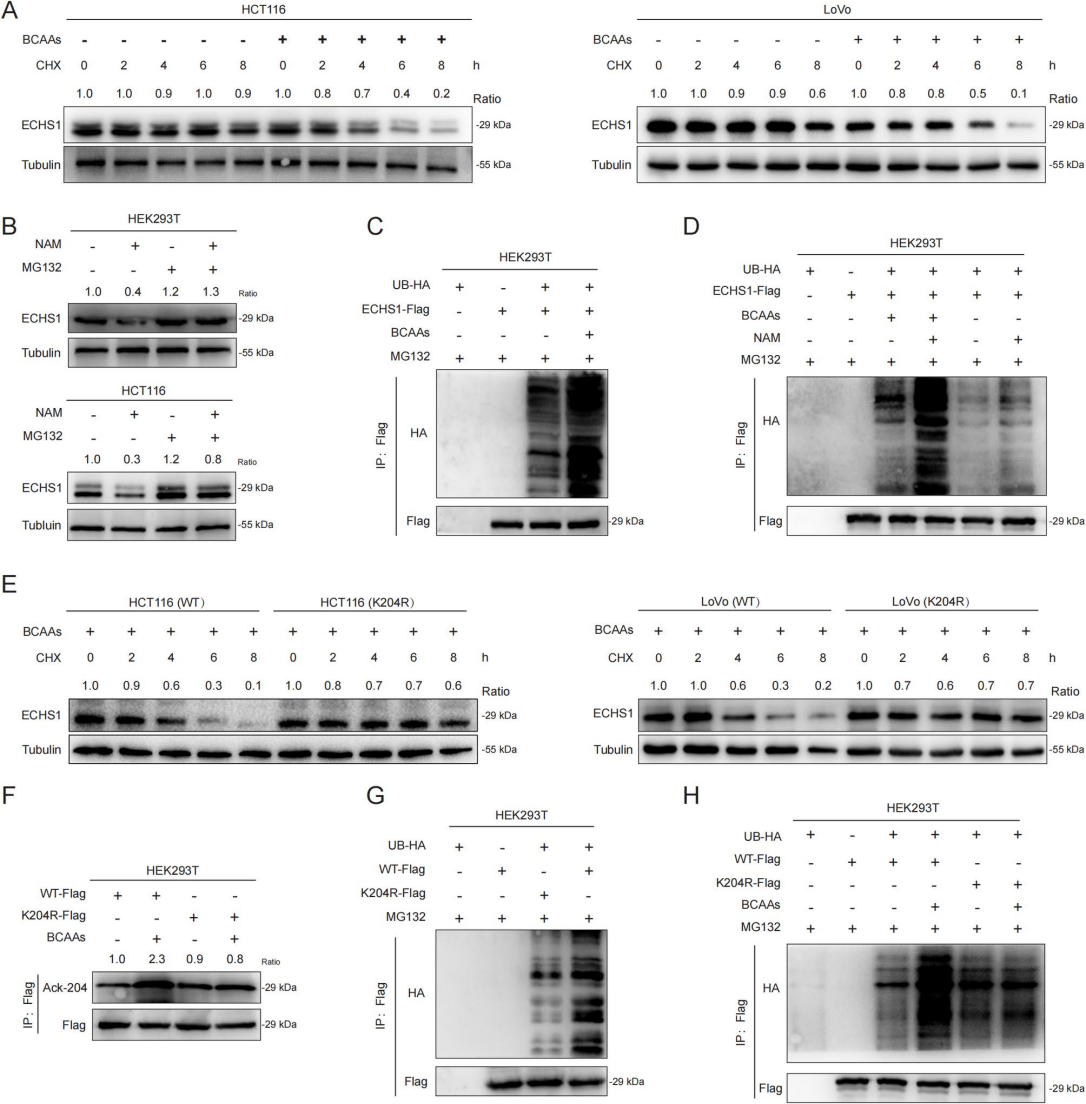
Acetylation-induced ubiquitination and degradation of ECHS1. **A** Effects of ECHS1 expression in HCT116 and LoVo cells cultured with or without BCAA-supplemented medium following CHX treatment. **B** Detection of ECHS1 protein expression levels in HEK293T and HCT116 cells treated with proteasome inhibitor MG132 and NAM. **C**, **D** Expression of ECHS1 protein ubiquitination levels under BCAA and MG132 treatment. **E** Comparison of protein stability between wild-type and K204R mutant ECHS1 following BCAA treatment via CHX. **F–H** Effects of BCAAs on the acetylation and ubiquitination of wild-type ECHS1 compared with those of the K204R mutant. ECHS1: Enoyl-CoA hydratase-1, CHX: Cycloheximide, BCAA: Branched-chain amino acids, NAM: Nicotinamide, HEK293T: Human embryonic kidney 293T cells, K204R: K204 arginine mutant

We further investigated how BCAAs reduce ECHS1 protein levels. In both HEK293T cells and KRAS-mutant CRC cells, BCAAs lowered the endogenous levels of the ECHS1 protein, whereas the ECHS1 mRNA levels were unaffected, suggesting posttranscriptional regulation of ECHS1 expression by BCAAs (Supplementary Fig. 4A, B). Cycloheximide (CHX) chase experiments revealed that BCAAs accelerated ECHS1 degradation in HCT116 and LoVo cells. In contrast, HT29 and SW48 cells exhibited no significant ECHS1 degradation with BCAA, underscoring KRAS-mutant specificity. (Fig. 5A; Supplementary Fig. 4C). The proteasome inhibitor MG132 blocked the NAM-mediated reduction in ECHS1 protein in HEK293T and HCT116 cells, suggesting that acetylation regulates ECHS1 degradation via the ubiquitin‒proteasome pathway (Fig. 5B). BCAAs increased ECHS1 ubiquitination, an effect that was further enhanced by NAM treatment (Fig. 5C, D). Following CHX treatment, the K204R mutant ECHS1 cells exposed to BCAAs presented greater protein stability than did the wild-type cells (Fig. 5E). BCAAs increased the acetylation and ubiquitination of wild-type ECHS1 but did not affect the K204R mutant (Fig. 5F-H). These results suggest that BCAAs promote ECHS1 ubiquitination and degradation via increased acetylation.

### CBP mediates the acetylation of K204 in ECHS1

**Fig. 6:**
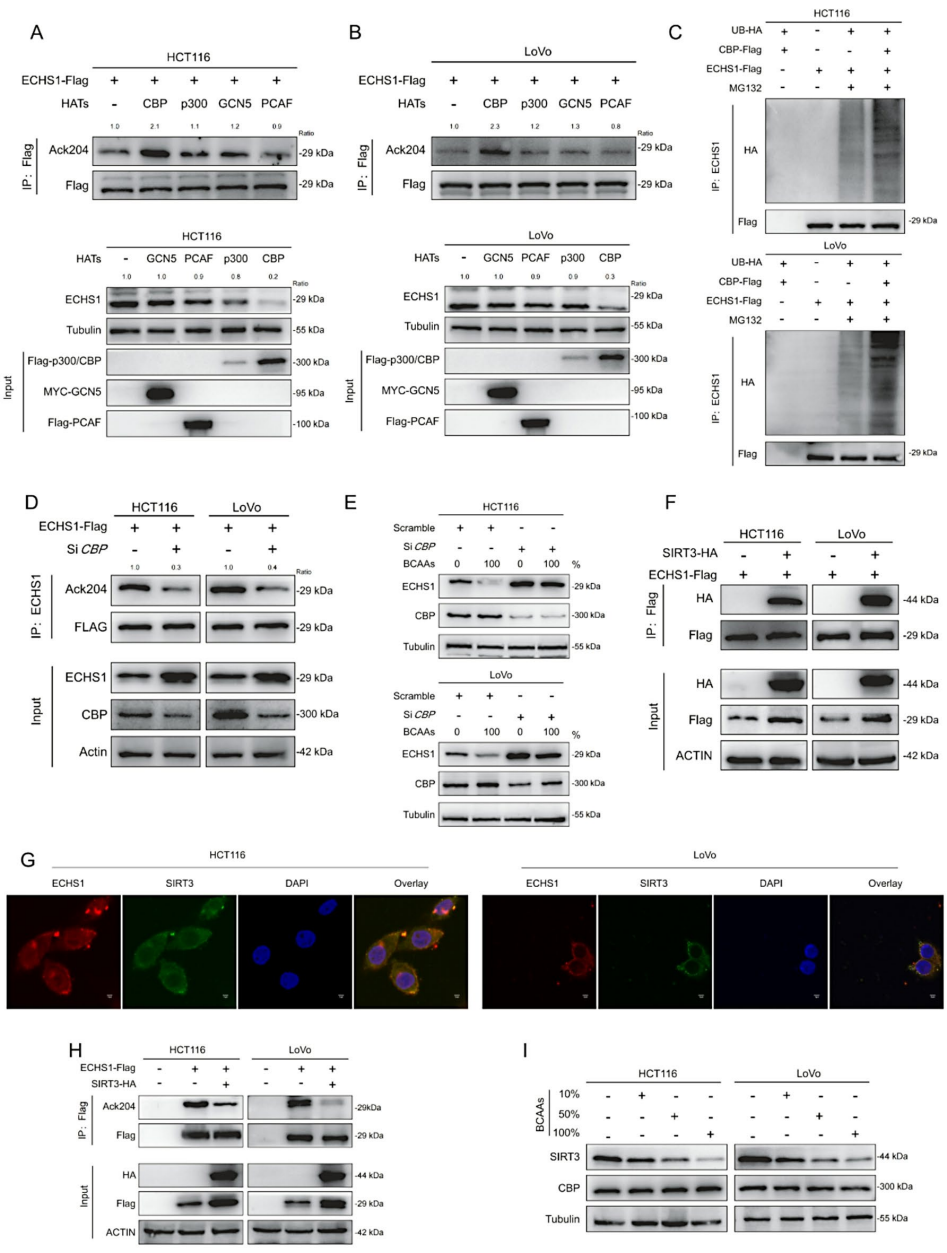
CBP and SIRT3 regulate the acetylation of ECHS1 at K204. **A**, **B** Effects of overexpressing acetyltransferases (p300, CBP, PCAF, and GCN5) on acetylation levels at K204 in HCT116 and LoVo cells. **C** Impact of CBP overexpression on ECHS1 ubiquitination and protein levels. **D** Effects of CBP inhibition on K204 acetylation levels and ECHS1 protein levels. **E** Counteraction of BCAA-induced reduction in ECHS1 by CBP inhibition. **F** Interaction between SIRT3 and ECHS1 in HCT116 and LoVo cells. **G** Colocalization of ECHS1 and SIRT3 in the cytoplasm. **H** Effect of SIRT3 overexpression on acetylation levels at AcK204. **I** Detection of SIRT3 and CBP expression levels in HCT116 and LoVo cells under BCAA treatment. CBP: CREB-binding protein, SIRT3: Sirtuin 3, ECHS1: Enoyl-CoA hydratase-1, K204: Lysine 204, p300: p300/CBP-associated factor, PCAF: P300/CBP-associated factor, GCN5: General control of amino acid synthesis 5, IP: Immunoprecipitation, BCAA: Branched-chain amino acids, IF: Immunofluorescence

To identify the acetyltransferase responsible for K204 acetylation in ECHS1, we transfected p300, CBP, PCAF, and GCN5 into HCT116 and LoVo cells. Compared with the other three acetyltransferases, only CBP overexpression significantly increased K204 acetylation (Fig. 6A, B). Furthermore, CBP overexpression markedly increased ECHS1 ubiquitination and decreased its protein level (Fig. 6C). Additionally, the inhibition of CBP expression significantly reduced K204 acetylation and increased ECHS1 protein levels (Fig. 6D). This effect was observed in both the endogenous protein levels of ECHS1 and in counteracting the BCAA-induced reduction in ECHS1 (Fig. 6E). These findings strongly indicate that CBP, the primary acetyltransferase for ECHS1, acetylates the K204 site and promotes its degradation.

### SIRT3 facilitates the deacetylation of K204 in ECHS1

Given the increased acetylation of ECHS1 induced by NAM, we hypothesized that SIRT family members may be involved in its deacetylation. SIRT3 is a major deacetylase in the SIRT family [32–34]. Therefore, we investigated whether SIRT3 could regulate ECHS1 deacetylation in the KRAS-mutant CRC cell lines HCT116 and LoVo. Immunoprecipitation experiments demonstrated an interaction between SIRT3 and ECHS1 (Fig. 6F). Immunofluorescence analysis revealed that ECHS1 and SIRT3 were primarily colocalized in the cytoplasm (Fig. 6G). SIRT3 overexpression significantly reduced AcK204 acetylation (Fig. 6H). Additionally, compared with CBP, BCAAs significantly decreased SIRT3 expression, which is consistent with previous findings [35] (Fig. 6I). In contrast, HT29 cells showed negligible BCAA effects on SIRT3 and CBP expression (Supplementary Fig. 5A), emphasizing KRAS-mutant specificity. These results suggest that BCAAs may cause excessive ECHS1 acetylation by downregulating SIRT3, leading to decreased ECHS1 levels.

### Synergistic Inhibition of KRAS-positive tumors and the role of BCAA metabolism in CRC

**Fig. 7:**
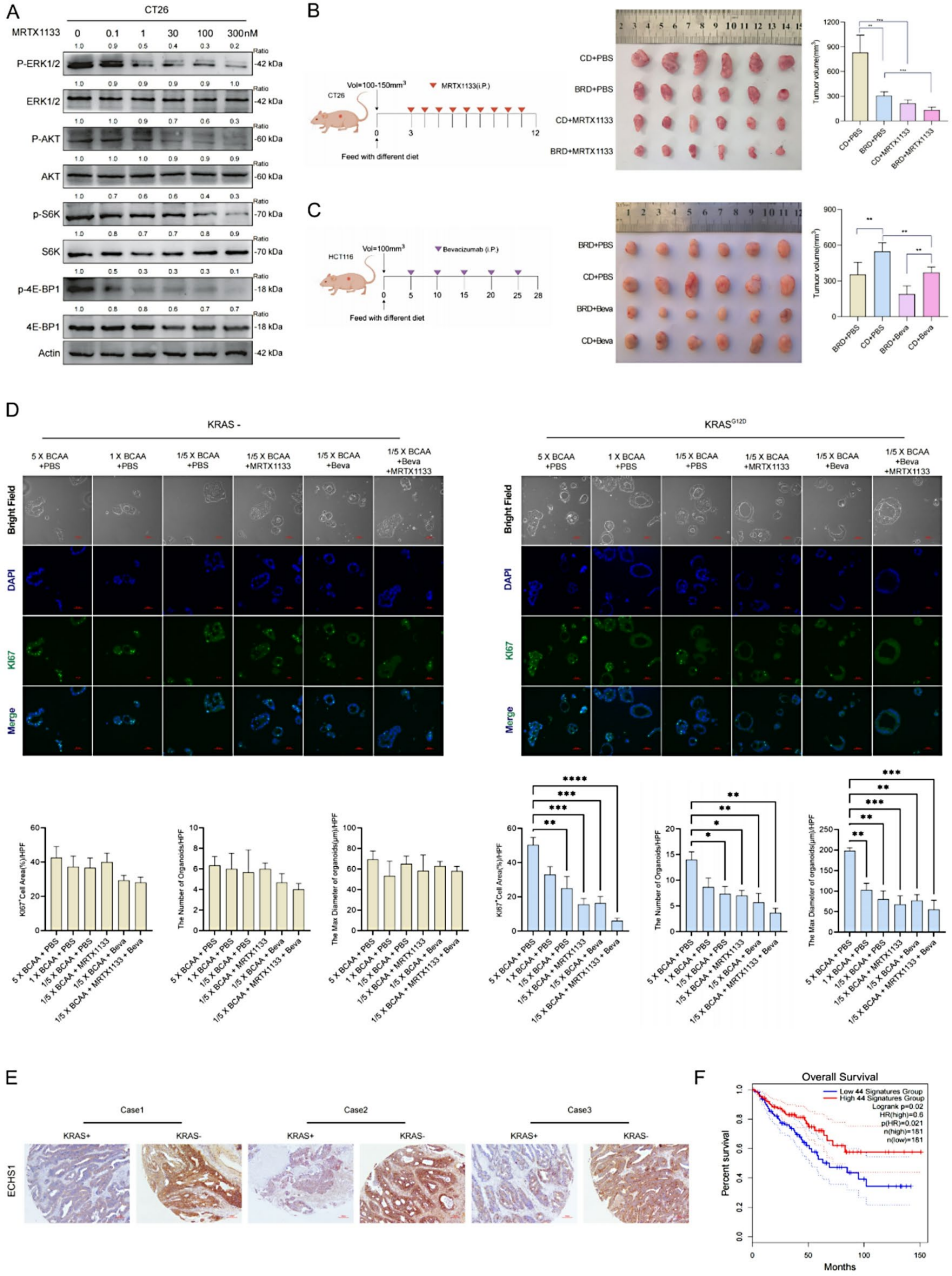
Synergistic inhibition of KRAS-positive tumors by BCAA restriction and targeted therapies. **A** Impact of MRTX1133 on the KRAS signaling pathway in the KRAS G12D-mutant CT26 cell lines. **B** Synergistic effects of MRTX1133 and BRD on subcutaneous CT26 tumor growth. **C** Synergistic effects of BRD and Beva on subcutaneous HCT116 tumor growth. **D** The proliferation levels of organoids under treatment with BCAA-rich condition, BCAA-restricted condition, Beva, MRTX1133, or their combination. **E** Analysis of ECHS1 expression in CRC tissue arrays categorized by KRAS gene status. **F** Correlations between expression of BCAA-metabolizing enzymes and patient survival outcomes. KRAS: Kirsten rat sarcoma viral oncogene homolog, G12D: G12D mutation (a specific mutation in KRAS), BRD: BCAA-restricted diet, BCAA: Branched-chain amino acids, Beva: Bevacizumab (an anti-VEGF antibody), ECHS1: Enoyl-CoA hydratase-1, MRTX1133: the KRAS G12D inhibitor, pERK: Phosphorylated-extracellular signal-regulated Kinase, pAKT: Phosphorylated-Protein Kinase B(PKB), pS6: Phosphorylated-Ribosomal Protein S6, p4EBP1: Phosphorylated-Eukaryotic Translation Initiation Factor 4E-Binding Protein 1

Studies have shown that MRTX1133 specifically targets the KRAS G12D mutation [36]. We evaluated its effects on MRTX1133 on the KRAS signaling pathway in the KRAS G12D-mutant CT26 and HCT116 cell lines. After 3-hour treatment, MRTX1133 inhibited key signaling molecules such as pERK, pAKT, pS6, and p4EBP1 in CT26 cells in a concentration-dependent manner (Fig. 7A). In HCT116 (KRAS G13D), MRTX1133 inhibited signaling with reduced efficacy compared to CT26 (Supplementary Fig. 5B), consistent with G12D specificity. The partial effect in HCT116 suggests mutation-specific responses.

In vivo, MRTX1133 reduced tumor volume in CT26 xenografts, with enhanced suppression when paired with BCAA restriction (Fig. 7B). While BCAA restriction synergized with bevacizumab to reduce HCT116 tumor growth via nutrient limitation (Fig. 7C), preliminary data with FOLFOX showed similar tumor suppression (Supplementary Fig. 5C). However, bevacizumab was prioritized for its alignment with BCAA metabolism. Western blot analysis revealed no additional modulation of mTORC1 signaling (pS6, p4EBP1) beyond FOLFOX’s cytotoxic effects (Supplementary Fig. 5D), unlike the metabolic specificity of BCAA restriction alone (Fig. 3B–C). This indicates FOLFOX’s efficacy is driven more by cytotoxicity than by enhancing BCAA-related pathways.

Bright-field analysis showed BCAA-rich diets boosted KRAS-G12D mutant organoid growth, reversed by BCAA restriction and enhanced by bevacizumab or MRTX1133 (Fig. 7D), unlike wild-type organoids. Morphometric data highlight synergistic therapeutic potential.

**Fig. 8:**
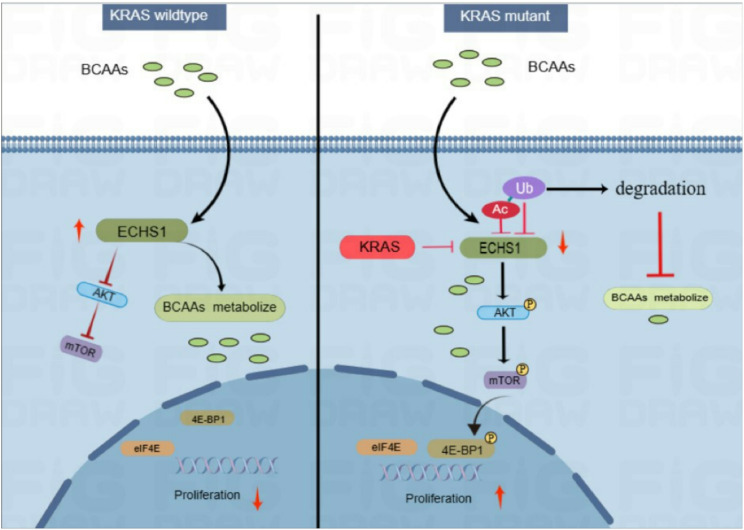
Proposed model of BCAA metabolism in KRAS-mutant CRC. KRAS mutations promote BCAA accumulation by enhancing acetylation and degradation of ECHS1, reducing BCAA catabolism and activating mTORC1 signaling

Further analysis of CRC tissue arrays, categorized by KRAS gene status, revealed a significant decrease in ECHS1 expression in patients with KRAS mutations (Fig. 7E). Low expression of BCAA-metabolizing enzymes was correlated with poorer patient survival outcomes (Fig. 7F). To summarize our findings, we propose a model in which KRAS mutations drive BCAA accumulation by promoting acetylation and degradation of ECHS1, leading to mTORC1 activation and CRC proliferation. (Fig. 8).

## Discussion

The metabolic reprogramming of cancer cells provides promising ground for identifying new therapeutic targets and understanding tumor biology [37, 38]. Our study provides crucial insights into the metabolic alterations induced by KRAS mutations in CRC, particularly through the dysregulation of BCAA metabolism. Acetylation-induced degradation of ECHS1 leading to the accumulation of BCAAs appears to be a significant driver of CRC proliferation in KRAS-mutant contexts.

Previous studies have highlighted the role of KRAS mutations in enhancing glycolysis and redirecting glycolytic intermediates to biosynthetic pathways essential for cancer cell proliferation [39, 40]. Consistent with these observations, our results extend the understanding of KRAS-driven metabolic reprogramming to include profound effects on amino acid metabolism. Notably, the loss of ECHS1 activity triggered by lysine acetylation and subsequent proteasomal degradation underpins a critical shift in BCAA catabolism. This mechanism contributes to the accumulation of BCAAs in KRAS-mutant CRC cells, further activating the mTORC1 signaling pathway, a well-known promoter of tumoral processes [41].

Our findings align with those of previous studies, which demonstrated that impaired BCAA metabolism could support the oncogenic potential of KRAS mutations in pancreatic cancer [19]. Similarly, our study underscores the potential of targeting BCAA metabolism as a therapeutic strategy for CRC harboring KRAS mutations. The restriction of dietary BCAAs, as shown in our in vivo models, notably inhibited tumor proliferation without affecting metastatic capacity, suggesting that dietary interventions could be an adjunctive strategy alongside conventional therapies. Furthermore, the observation that BCAA restriction does not impair metastatic capabilities invites a deeper exploration of the distinct metabolic requirements for tumor proliferation and metastasis. These findings suggest that while BCAA metabolism is crucial for cell proliferation, other pathways may sustain the metastatic phenotype. This distinction is critical for the design of targeted therapies that concurrently inhibit growth and spread.

The selective vulnerability of KRAS-mutant cells to BCAA depletion highlights the possibility of using metabolic targeting as a therapeutic strategy. Importantly, our study provides a basis for the enhanced efficacy of KRAS inhibitors when combined with dietary manipulation of BCAA levels. This synergy could overcome the intrinsic resistance observed in many KRAS-mutant cancers, which aligns with recent clinical investigations that suggest that metabolic interventions may increase the efficacy of targeted therapies [42].

The prognostic implications of ECHS1 expression levels in our cohort, with lower levels correlating with reduced survival, are clinically relevant. These findings suggest that ECHS1 could serve not only as a therapeutic target but also as a potential biomarker for aggressive KRAS-mutant CRC. This is supported by our comprehensive analysis of ECHS1 downregulation across multiple patient samples, reinforcing its role in metabolic alterations associated with poor outcomes. Building on this, future screening of AcK204 in CRC samples will explore its potential as a biomarker, leveraging its established mechanistic role in ECHS1 acetylation and degradation to further enhance its clinical utility.

In conclusion, our findings delineate a critical role for BCAA metabolism reprogramming in KRAS-mutant CRC which is mediated through the acetylation-dependent degradation of ECHS1. This pathway not only advances our understanding of the metabolic underpinnings of cancer proliferation but also highlights novel targets for therapeutic intervention. As we move forward, it is imperative that future research aims to translate these insights into clinical trials that rigorously test the efficacy of combining BCAA restriction with targeted therapies. Moreover, investigating the broader metabolic networks and their interactions with oncogenic pathways in KRAS-mutant cancers will be essential to fully harness the potential of metabolic interventions. By concentrating on these areas, we can more effectively develop strategies that both suppress tumor growth and tackle the complexities of metastasis and drug resistance, ultimately contributing to enhanced outcomes for patients afflicted with this formidable malignancy.

## Materials and methods

### Cell lines and clinical samples

CRC cell lines, including SW48, HT29, LoVo, HCT116, DLD1, SW1116, SW480, SW620, CT26, MC38, and Caco2, were acquired from the American Type Culture Collection (ATCC). The KRAS G12D mutation was introduced into wild-type SW48 and HT29 cells using CRISPR/Cas9 technology. Briefly, the sgRNA targeting KRAS codon 12 was cloned into pSpCas9(BB)-2 A-Puro (PX459, Addgene, #62988) and transfected using Lipofectamine 3000 (Invitrogen, #L3000015) with 2 µg plasmid DNA per 10⁶ cells. Stable clones expressing KRAS G12D were selected using puromycin, and the successful introduction of the mutation was confirmed by Sanger sequencing. Elevated KRAS protein expression was further validated by Western blot analysis. Cells were maintained in DMEM (Dulbecco’s Modified Eagle Medium, Gibco, #11965092) supplemented with 10% FBS (fetal bovine serum, Gibco, #10099141) and 1% penicillin/streptomycin (Gibco, #15140122) at 37 °C in a 5% CO₂ humidified incubator. All the cell lines were authenticated via short tandem repeat (STR) analysis, and they were confirmed to be free from mycoplasma contamination. A total of 120 paired samples comprising human CRC tissues and adjacent noncancerous tissues (NCTs) were obtained from Nanfang Hospital. Written consent was obtained from all the subjects, with approval from the relevant institutional review boards. This study adhered to the ethical standards set forth in the Declaration of Helsinki and the CIOMS International Ethical Guidelines for Biomedical Research Involving Human Subjects.

### Reagents and antibodies

The commercial antibodies used in this study and their manufacturers were as follows: anti-beta actin (Cat No. 66009-1-Ig, Proteintech), anti-beta-tubulin (Cat No. 66240-1-Ig, Proteintech), anti-DBT (Cat No. 12451-1-AP, Proteintech), anti-OXCT1 (Cat No. 12175-1-AP, Proteintech), anti-BCKDK (Cat No. 15718-1-AP, Proteintech), anti-ECHS1 (A3845, ABclonal; 11305-1-AP, Proteintech), anti-KRAS (ab191595, Abcam), anti-phospho-AKT (Ser473, #4060, CST), anti-phospho-S6K (Thr389,#9205,CST), anti-S6K (#9202,CST), Akt Antibody (#9272,CST), SIRT3 (AF5135, Affinity), anti-Ki67 (#9449,CST), anti-PCNA (#13110,CST), anti-e4E-BP1 (9452,CST), phospho-4E-BP1 (Thr37/46, #2855), DYKDDDDDDK tag polyclonal antibodies (14793,CST), Mouse Anti-rabbit IgG (Conformation Specific, 5127, CST), MRTX1133 and bevacizumab were purchased from Selleck, NAM (72345, Sigma), MA132(M8699, Sigma), Cycloheximide(CHX, 239763, Sigma). Primary antibodies were used at the following dilutions for Western blot: anti-ECHS1 (A3845, ABclonal, 1:1000), anti-KRAS (ab191595, Abcam, 1:500), and anti-beta actin (66009-1-Ig, Proteintech, 1:5000).

### Mice and diet

Male athymic nude mice (BALB/c-nu/nu, 6 weeks old) purchased from the Animal Center of Guangdong Province were used to construct subcutaneous xenograft models. HCT116, HT29, CT26, and MC38 cell lines (2 × 10⁶ cells per mouse) were resuspended in 100 µL of PBS and subcutaneously injected into the right flank of each mouse using a 1 mL syringe with a 26-gauge needle. Tumor growth was monitored starting 6–7 days post-injection, when tumors became visible to the naked eye. Tumor volume was measured every 3 days using the formula: Volume = length × width × width / 2. The starting time for measurement varied depending on the cell line, with measurements taken accordingly. Tumor measurements began when tumors reached approximately 50–100 mm³ (typically 6–7 days post-injection), recorded every 3 days using digital calipers. MRTX1133 (10 mg/kg, i.p., daily) and bevacizumab (5 mg/kg, i.p., twice weekly) [43] were administered with CD or BRD for 3 weeks. Diet compositions were detailed in Supplementary Tables S2 and S3. Mouse weight and adverse effects were monitored, with data detailed in Supplementary Tables S4, S5 and S6. All animal care and experiments were approved by the Institutional Animal Care and Use Committee (IACUC) of Nanfang Hospital. All animal studies complied with relevant ethical regulations for animal testing and research. The mice were fed either a control diet (CD) or the BCAA-restricted diet (BRD). Diets were purchased from the Dyets Company.

### Quantification of BCAA concentrations

BCAA concentrations in the culture medium were quantified via a BCAA kit (Sigma, MAK003) following the manufacturer’s instructions. Twenty-four hours postcultivation on a 10 cm plate, the medium was collected, and BCAAs were measured in a 100 µl reaction mixture. The absorbance at 450 nm was recorded to determine the BCAA concentration. The cells were subsequently washed, detached, and counted for normalization.

### CCK8 and colony formation assays

For cell proliferation assays, cells (1,000 cells per well) were seeded in 96-well plates, and their viability was assessed daily for 6–7 consecutive days using the CCK8 assay. Specifically, 10 µL of CCK8 reagent (Dojindo Laboratories, Japan, #CK04) was added per well, incubated for 2 h at 37 °C, and absorbance was measured at 450 nm with a SpectraMax i3x (Molecular Devices). For the colony formation assays, cells (500 cells per well) were seeded in 6-well plates and cultured in DMEM supplemented with 10% FBS at 37 °C in a 5% CO₂ humidified incubator for 8–12 days. At the end of the experiment, colonies were washed with PBS, fixed in methanol, and stained with 0.1% crystal violet. Colonies exceeding 50 cells per well were counted in triplicate.

### EdU assay

Cell proliferation was evaluated using a 5-ethynyl-2’-deoxyuridine (EdU) assay kit (RiboBio, Guangzhou, China, #C10310-1). Cells were seeded at a density of 3,000 cells per well in 96-well plates and cultured in DMEM supplemented with 10% FBS and 1% penicillin/streptomycin at 37 °C in a 5% CO₂ humidified incubator. After 72 h of incubation, cells were treated with 50 µM EdU (prepared by diluting the stock solution in culture medium according to the manufacturer’s instructions) and incubated for 2 h at 37 °C. Following this, cells were fixed with 4% formaldehyde (Sigma, #F8775) in PBS for 30 min at room temperature, then permeabilized with 0.1% Triton X-100 (Sigma, #X100) in PBS for 20 min. The EdU detection solution (100 µL per well, containing Alexa Fluor 488 azide) was added to the culture medium and incubated for 30 min at room temperature in the dark, per the kit protocol. Nuclear staining was performed by adding 100 µL of Hoechst 33,342 (1:1000 dilution in PBS, RiboBio) and incubating for 10 min at room temperature. After washing three times with PBS (5 min each), fluorescence was visualized using a fluorescence microscope (Nikon Eclipse Ti2) with excitation/emission wavelengths of 495/519 nm for EdU (green) and 350/461 nm for Hoechst (blue). Images were captured and analyzed for EdU-positive cells in triplicate experiments.

### Immunoprecipitation and Western blotting

For immunoprecipitation (IP), cells were harvested, washed twice with ice-cold PBS, and lysed in a lysis buffer containing 50 mM Tris-HCl (pH 7.5, Sigma, #T3253), 150 mM NaCl, 0.5% Nonidet P-40 (NP-40, Sigma, #74385), and 1× protease inhibitor cocktail (Sigma-Aldrich, #P8340, diluted 1:100 from stock), unless otherwise specified. Approximately 1 × 10⁷ cells were lysed in 1 mL of buffer on ice for 30 min with intermittent vortexing. Lysates were centrifuged at 12,000 rpm for 15 min at 4 °C using a refrigerated centrifuge, and the supernatant was collected. For FLAG-tagged protein IP, 500 µg of protein (quantified by BCA assay, Thermo Fisher, #23225) was incubated with 20 µL of anti-FLAG M2 agarose beads (Sigma-Aldrich, #A2220) in a 1.5 mL microcentrifuge tube for 3 h at 4 °C with gentle rotation (10 rpm) on a rotary mixer. Alternatively, for specific antibody IP, lysates were incubated with primary antibodies (e.g., anti-ECHS1, ABclonal, A3845, 2 µg) and 20 µL of Protein G agarose beads (Thermo Fisher, #20398). Beads were washed three times with 1 mL of lysis buffer (5 min per wash at 4 °C), pelleted by centrifugation at 3,000 rpm for 1 min, and resuspended in 40 µL of 2× SDS loading buffer (125 mM Tris-HCl pH 6.8, 4% SDS, 20% glycerol, 10% β-mercaptoethanol, 0.02% bromophenol blue). Samples were boiled at 95 °C for 5 min using a heat block and cooled on ice.

For co-immunoprecipitation (co-IP) experiments, cells were lysed in a milder buffer containing 50 mM Tris-HCl (pH 7.5), 150 mM NaCl, 0.1% NP-40, and 1× protease inhibitor cocktail, prepared fresh and chilled to 4 °C. Approximately 2 × 10⁷ cells were lysed in 1 mL of buffer, and after centrifugation as above, the supernatant (1 mg protein) was incubated with anti-ECHS1 antibody (ABclonal, A3845, 2 µg) and 30 µL Protein G agarose beads for 4–5 h at 4 °C with rotation. Beads were washed three times with 1 mL of co-IP lysis buffer and processed as described for standard IP.

For Western blotting, extracted proteins or IP eluates were separated by SDS-PAGE using 10-12% polyacrylamide gels at 120 V for 90 min. Proteins were transferred to a PVDF membrane using a wet transfer system (Bio-Rad, #1703930) at 100 V for 1 h in transfer buffer (25 mM Tris, 192 mM glycine, 20% methanol). Membranes were blocked with 5% skim milk (Sigma, #70166) in TBST (Tris-buffered saline with 0.1% Tween-20, 20 mM Tris-HCl pH 7.5, 150 mM NaCl, 0.1% Tween-20) for 1 h at room temperature with gentle shaking. Primary antibodies were diluted in blocking buffer and incubated with membranes overnight at 4 °C with rocking. Membranes were washed three times with TBST (10 min each), then incubated with HRP-conjugated secondary antibodies in TBST with 5% milk for 1 h at room temperature. After three additional TBST washes (10 min each), protein bands were visualized using an enhanced chemiluminescence (ECL) kit (Vazyme, E412-02) and imaged with a ChemiDoc Imaging System (Bio-Rad). All experiments were performed in triplicate.

### Immunohistochemistry (IHC) staining and scoring

For immunohistochemical staining of human CRC tissues, paraffin-embedded sections were first baked at 65 °C for 1–2 h. The tissues were then deparaffinized by placing them in three jars of xylene for 10 min each, followed by rehydration through a graded alcohol series (100% ethanol 1, 100% ethanol 2, 95% ethanol, 90% ethanol, 80% ethanol, and 70% ethanol), with each step lasting 1 min. After rehydration, the sections were washed three times in deionized water (ddH2O) for 1 min each. Antigen retrieval was performed using citric acid or EGTA, and endogenous peroxidase activity was blocked by incubating the sections with a peroxidase blocking agent for 10–15 min, followed by three washes with PBS. To block non-specific binding, the sections were incubated with goat serum working solution at room temperature for 1 h, and then washed once with PBS. Human CRC tissues were stained with antibodies against ECHS1(1:400). Paraffin-embedded tissues were sectioned at 4 μm and incubated with the appropriate antibodies at 4 °C overnight. The subsequent steps followed the protocols provided by the GTVision III Detection System/Mo&Rb manufacturer (Gene Tech, China). Additionally, paraffin-embedded xenograft tissue sections were stained with anti-Ki67(1:800), anti-ECHS1(1:400), and anti-PCNA (1:800) antibodies. The staining results were evaluated by two independent authors who were blinded to the patients’ clinicopathological data. Score 0: no staining or incomplete and faint/barely perceptible cytoplasmic staining in ≤ 10% of tumor cells; Score 1: incomplete and faint/barely perceptible cytoplasmic staining in > 10% of tumor cells; Score 2: weak-moderate complete cytoplasmic staining in > 10% of tumor cells OR intense cytoplasmic staining in ≤ 10% of tumor cells; Score 3: complete and intense membrane staining in > 10% of tumor cells.

### Expression and purification of Recombinant human ECHS1

The mutants pRH281-ECHS1 K101R, K118R, K204R, and K204Q were constructed via a site-directed mutagenesis kit (C215-01, Vazyme, China). Protein expression was achieved in *Escherichia coli* BL21 (DE3) cells. A single colony was isolated, propagated in LB medium supplemented with ampicillin (100 mg/L), and incubated at 37 °C with shaking at 200 rpm. Protein induction was triggered at 18 °C using 400 µmol/L 3-indoleacrylic acid upon reaching an optical density of 0.4–0.6 at 600 nm (OD600). Following a 10-hour induction period, the cells were collected and disrupted. The lysates were centrifuged at 12,000 rpm for 30 min, and the supernatants were subsequently incubated with His-tag affinity beads for 5 h at 4 °C. The proteins were eluted with a 6× His peptide at 4 °C for 2 h and concentrated via ultrafiltration.

### Immunofluorescence (IF) analyses

For cell fluorescence, cells were pre-seeded on 24-well plates with coverslips (biosharp, BS-14-RC, 14 mm) at a density of 100,000 target cells per well. After appropriate treatments, the cells were fixed according to established protocols and incubated with primary antibodies at a 1:100 dilution. Then, they were treated with fluorescent dye-conjugated secondary antibodies and stained with DAPI.

For tissue fluorescence, tissue preparation (repair and blocking) followed the same protocol as immunohistochemistry (IHC). Primary antibodies were added at a 1:100 dilution and incubated overnight at 4 °C. The next day, the samples were warmed to room temperature for 30 min, washed three times with TBST, and incubated with corresponding secondary antibodies at a 1:400 dilution for 1 h at room temperature. After three washes with TBST, fluorescent antibodies were applied and incubated for 30 min at room temperature. After another three washes with TBST, DAPI working solution was added and incubated for 5 min at room temperature. The tissues were then washed three times with TBST, and an anti-fluorescence quencher was applied before the slides were sealed with a coverslip.

For multicolor cell fluorescence, the same procedure was used with cell coverslips. After the first round of staining, microwave antigen retrieval was performed, and the same staining steps were repeated. Finally, DAPI was added and the slides were sealed with a coverslip.

### Identification of ECHS1 acetylation sites

HEK293T cells were transfected with FLAG-tagged ECHS1 and incubated for 48 h. Prior to harvesting, the cells were treated with 5 mM nicotinamide (NAM, 72345, Sigma, USA) for 6 h. Total cell lysates were then incubated with Flag (M2) magnetic beads for more than 4 h, followed by three washes with ice-cold NP40 buffer. Proteins were eluted from the beads by incubation with a 3× Flag peptide mixture (P9801, Beyotime) at 4 °C for 2 h. The eluted proteins were separated via SDS‒PAGE and stained with Coomassie Brilliant Blue. Protein bands at 21–34 kDa were excised and analyzed by mass spectrometry via PTM Biolabs (Shenzhen, China).

### Mass spectrometry analyses

Total lysates from HEK293T cells transfected with ECHS1-Flag were incubated with anti-FLAG M2 magnetic beads for over 4 h at 4 °C. The protein-bead complexes were washed three times with ice-cold NP40 buffer. Proteins were subsequently eluted from the beads by incubation with a 3× FLAG peptide solution (P9801, Beyotime, China) at 4 °C for 2 h. The eluted proteins were then subjected to mass spectrometry analysis via PTM Biolabs (Shenzhen, China).

### Measurement of ECHS1 activity

ECHS1 enzyme activity was measured via previously established methods. The assay involved adding ECHS1 to a reaction mixture containing 130 µl of 50 mM Tris-HCl (pH 8.0) and 0.25 mM crotonyl-CoA. The reaction progress was monitored by quantifying the decrease in absorbance at 263 nm.

### SiRNA transfection and RNA interference

CBP expression is reduced by RNA interference. Synthetic siRNA oligonucleotides were sourced from Shanghai Gene Pharma Co., Ltd., employing the following sequences: siCBP-1, 5′-GGAAGCAGCUGUGUACCAUTTdTdT-3′; siCBP-2, 5′-GCAUGAAUGCUAACUUUAAdTdT-3′; and siCBP-3, 5′-CCUACAGAUAUCAAGAAUAdTdT-3. Transfection of each siRNA was carried out via Lipofectamine RNAiMAX (Invitrogen, Thermo Fisher Scientific) according to the manufacturer’s protocol. The knockdown efficacy was confirmed by western blot analysis.

### Ubiquitinylation assay

For the ubiquitinylation analysis, the protease inhibitor MG132(100µmol, Sigma) was administered 4 h before the cells were harvested. The cells were then harvested and lysed in 1% SDS buffer (Tris-HCl pH 7.5, 0.5 mM EDTA, and 1 mM DTT), followed by boiling for 10 min post lysis. Immunoprecipitation of the lysed proteins was performed by adding antibodies to the lysate, which was diluted tenfold with 10 mM Tris-HCl buffer.

### Statistical analyses

The experiments were independently performed three times, yielding similar results. Statistical analyses were conducted via an unpaired two-tailed Student’s t test in GraphPad Prism 8.0. The data are presented as the mean ± standard deviation (S.D.). Statistically significant differences are denoted by the p values: *p < 0.05, **p < 0.01, ***p < 0.001, and ****p < 0.0001.

## Electronic supplementary material

Below is the link to the electronic supplementary material.

Supplementary Figure S1. Effects of BCAA restriction on KRAS-mutant CRC cell migration and metastasis. A, B Wound healing and Traswell assays in KRAS-mutant HCT116 and SW620 cells subjected to CD or BRD conditions. C, D Quantification of spleen-to-liver metastatic tumor weights in the HCT116 and CT26 models. The data represent the means ± SDs. SD: Standard deviation.; ns, not significant. KRAS: Kirsten rat sarcoma viral oncogene homolog, BCAA: Branched-chain amino acids, CD: Control diet, BRD: BCAA-restricted diet.

Supplementary Figure S2. Expression levels and regulatory effects of ECHS1 in KRAS-mutant CRC cells. A, B Quantitative RT‒PCR analysis of the mRNA expression of BCAA metabolism-related genes across various CRC cell lines (SW48, Caco2, HCT116, SW620). C, D Effect of KRAS knockdown or overexpression on KRAS mRNA and protein expression in HCT116 and LoVo cells. E EdU incorporation assay detected the proliferation in HCT116 and LoVo cells after KRAS knockdown. F CCK8 assay detected the proliferation under different BCAA conditions in HCT116 and LoVo cells. RT-PCR: Reverse transcription polymerase chain reaction, mRNA: Messenger RNA, ECHS1: Enoyl-CoA hydratase-1, ECHS1-OE: ECHS1 overexpression, Lv-con: Lentiviral control, KRAS: Kirsten rat sarcoma viral oncogene homolog, EdU incorporation assay: 5-Ethynyl-2’-deoxyuridine incorporation assay, CCK8 assay: Cell counting Kit-8 assay.

Supplementary Figure S3. Acetylation of ECHS1 in response to BCAA levels and NAM treatment in KRAS-mutant CRC cells. A Western blot analysis of ECHS1 expression under different BCAAs condition and KRAS status. B Western blot analysis of ECHS1 acetylation in LoVo cells transfected with ECHS1-Flag, treated with NAM (5, 10, 15 mM for 5 h; 10 mM for 2, 4, 6, 8 h). C ECHS1 acetylation in HCT116 cells transfected with ECHS1-Flag and treated with the deacetylase inhibitors NAM and TSA. D Relative ECHS1 enzymatic activity in LoVo cells transfected with wild-type (WT) ECHS1, K204Q (K/Q), or K204R (K/R) mutants. ECHS1: Enoyl-CoA hydratase-1, NAM: Nicotinamide, TSA: Trichostatin A, WT: Wild-type, G12D: G12D mutation (a specific mutation in KRAS), K204Q: K204 glutamine mutant, K204R: K204 arginine mutant, K/Q: K204Q mutant, K/R: K204R mutant.

Supplementary Figure S4. BCAA-induced posttranscriptional regulation and stability of the ECHS1 protein in CRC cells. A Quantitative RT‒PCR analysis the ECHS1 mRNA level. B Western blot analysis of ECHS1 protein stability in multiple cell lines (HEK293T, HCT116, LoVo, SW620, and DLD1). C Western blot analysis of ECHS1 protein levels in HT29 and SW48 cells treated with CHX at different time points (0, 2, 4, 6, and 8 h) after BCAA exposure. The data are presented as the means ± SDs; ns, not significant, SD: Standard deviation. ECHS1: Enoyl-CoA hydratase-1, BCAA: Branched-chain amino acids, RT-PCR: Reverse transcription polymerase chain reaction, mRNA: Messenger RNA, CHX: cycloheximide.

Supplementary Figure S5. Additional Analyses of BCAA, MRTX1133, and FOLFOX Effects in CRC Models. A Western blot analysis of SIRT3 and CBP expression in KRAS wild-type HT29 cells treated with BCAA (control vs. BCAA+). B Western blot analysis of pERK, pAKT, pS6, and p4EBP1 inhibition by MRTX1133 (0–300 µM, 3 h) in KRAS G13D-mutant HCT116 cells. C Tumor volume in HCT116 xenografts treated with BCAA restriction, FOLFOX (oxaliplatin 5 mg/kg, 5-FU 50 mg/kg), or their combination for 3 weeks. D Western blot analysis of mTORC1 pathway in HCT116 tumors. ECHS1: Enoyl-CoA hydratase-1, BCAA: Branched-chain amino acids, KRAS: Kirsten rat sarcoma viral oncogene homolog, MRTX1133: the KRAS G12D inhibitor. pERK: Phosphorylated-extracellular signal-regulated Kinase, pAKT: Phosphorylated-Protein Kinase B(PKB), pS6: Phosphorylated-Ribosomal Protein S6, p4EBP1: Phosphorylated-Eukaryotic Translation Initiation Factor 4E-Binding Protein 1.

Supplementary Material 6

Supplementary Material 7

Supplementary Material 8

Supplementary Material 9

Supplementary Material 10

Supplementary Material 11

## Data Availability

All data utilized and/or analyzed in this study are available from the corresponding authors, Zhiyong Shen or Haijun Deng, upon reasonable request.
