# Supplementary figures and images for: Acetylation-induced degradation of ECHS1 enhances BCAA accumulation and proliferation in KRAS-mutant colorectal cancer
Source: J Exp Clin Cancer Res. 2025 May 28;44:164. doi: 10.1186/s13046-025-03399-3 (PMC12117712; doi:10.1186/s13046-025-03399-3)

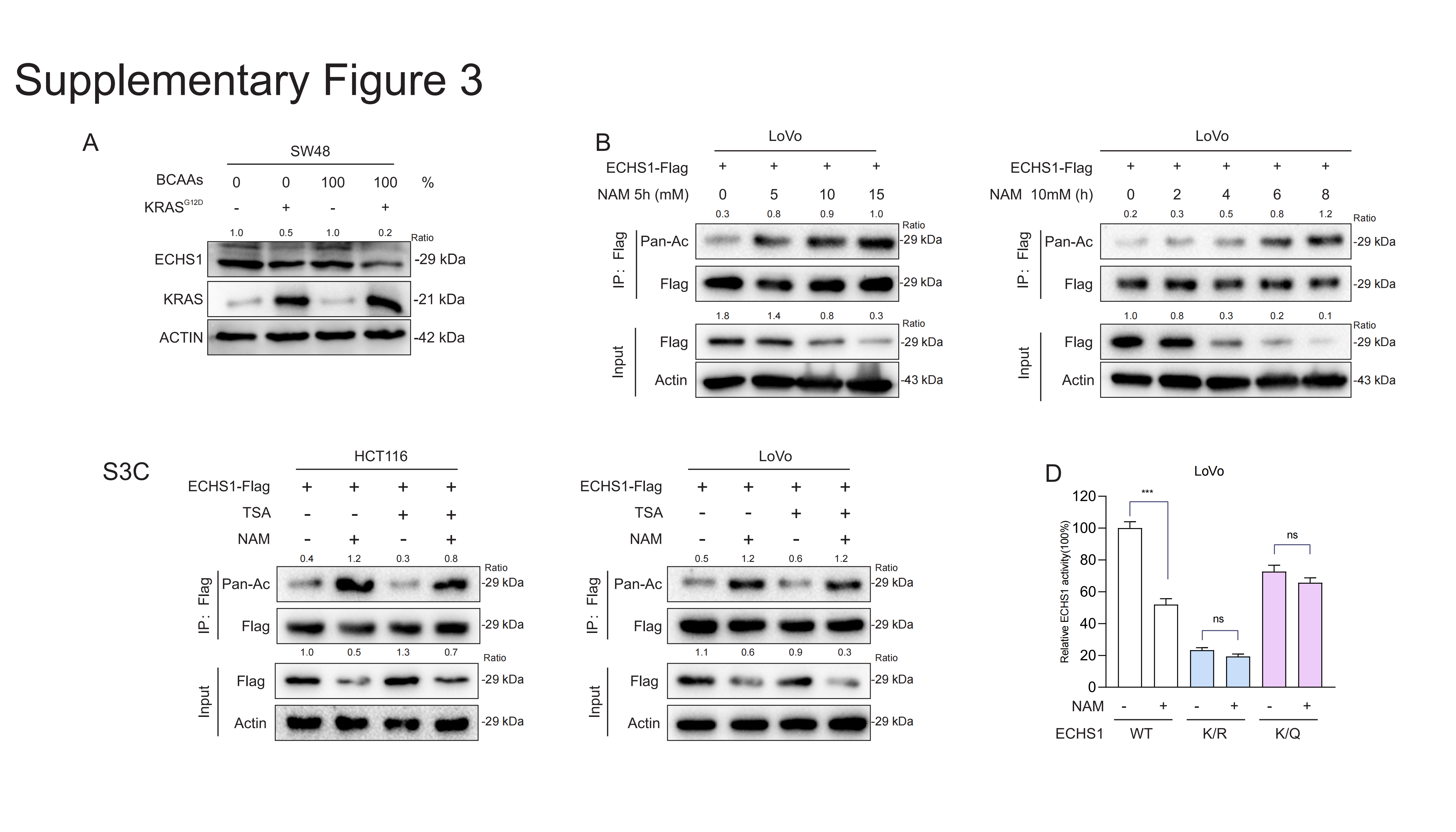

Supplement: Supplementary file 3 — Supplementary Figure S3. Acetylation of ECHS1 in response to BCAA levels and NAM treatment in KRAS-mutant CRC cells. A Western blot analysis of ECHS1 expression under different BCAAs condition and KRAS status. B Western blot analysis of ECHS1 acetylation in LoVo cells transfected with ECHS1-Flag, treated with NAM (5, 10, 15 mM for 5 h; 10 mM for 2, 4, 6, 8 h). C ECHS1 acetylation in HCT116 cells transfected with ECHS1-Flag and treated with the deacetylase inhibitors NAM and TSA. D Relative ECHS1 enzymatic activity in LoVo cells transfected with wild-type (WT) ECHS1, K204Q (K/Q), or K204R (K/R) mutants. ECHS1: Enoyl-CoA hydratase-1, NAM: Nicotinamide, TSA: Trichostatin A, WT: Wild-type, G12D: G12D mutation (a specific mutation in KRAS), K204Q: K204 glutamine mutant, K204R: K204 arginine mutant, K/Q: K204Q mutant, K/R: K204R mutant. [file 13046_2025_3399_MOESM3_ESM.tif]

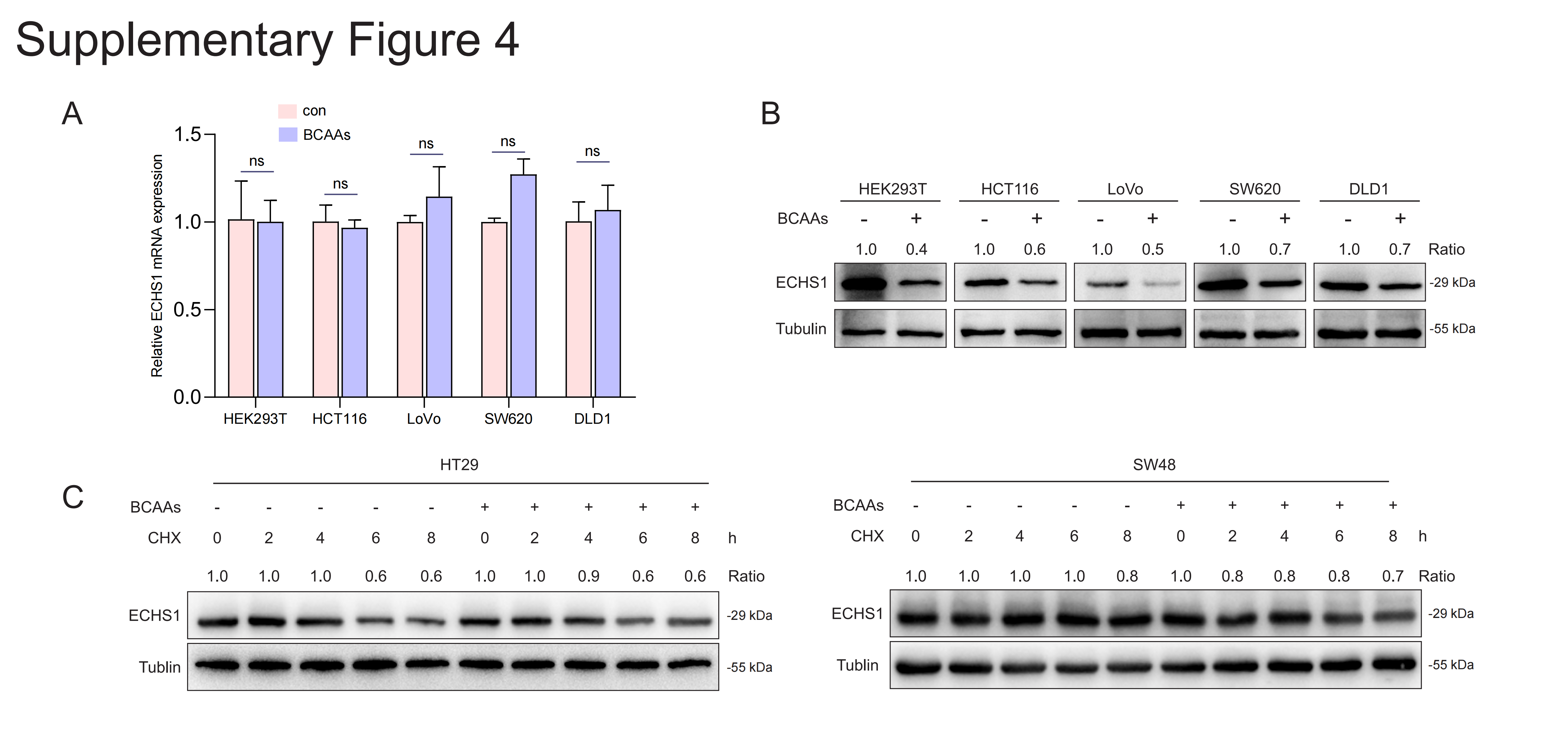

Supplement: Supplementary file 4 — Supplementary Figure S4. BCAA-induced posttranscriptional regulation and stability of the ECHS1 protein in CRC cells. A Quantitative RT‒PCR analysis the ECHS1 mRNA level. B Western blot analysis of ECHS1 protein stability in multiple cell lines (HEK293T, HCT116, LoVo, SW620, and DLD1). C Western blot analysis of ECHS1 protein levels in HT29 and SW48 cells treated with CHX at different time points (0, 2, 4, 6, and 8 h) after BCAA exposure. The data are presented as the means ± SDs; ns, not significant, SD: Standard deviation. ECHS1: Enoyl-CoA hydratase-1, BCAA: Branched-chain amino acids, RT-PCR: Reverse transcription polymerase chain reaction, mRNA: Messenger RNA, CHX: cycloheximide. [file 13046_2025_3399_MOESM4_ESM.tif]

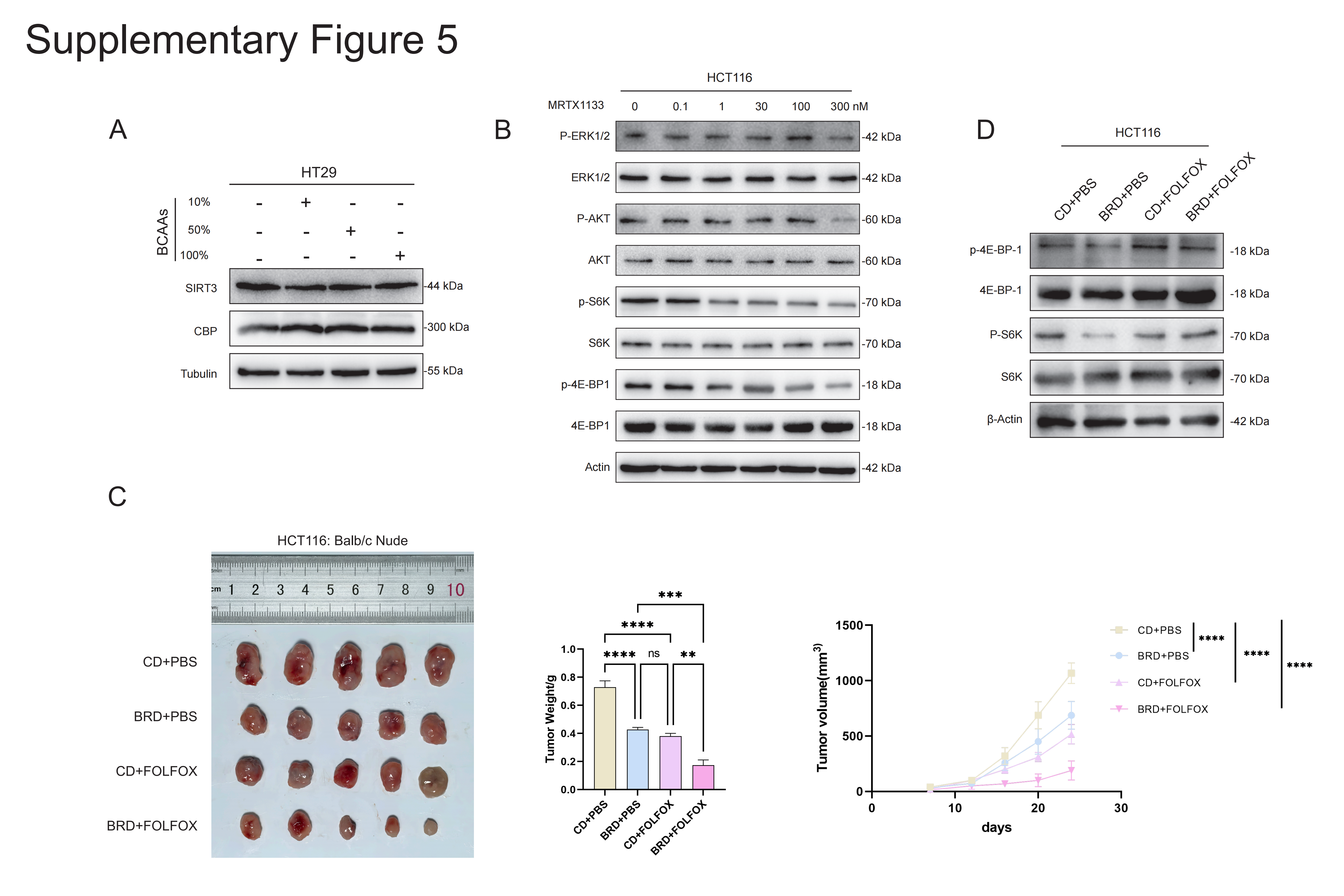

Supplement: Supplementary file 5 — Supplementary Figure S5. Additional Analyses of BCAA, MRTX1133, and FOLFOX Effects in CRC Models. A Western blot analysis of SIRT3 and CBP expression in KRAS wild-type HT29 cells treated with BCAA (control vs. BCAA+). B Western blot analysis of pERK, pAKT, pS6, and p4EBP1 inhibition by MRTX1133 (0–300 µM, 3 h) in KRAS G13D-mutant HCT116 cells. C Tumor volume in HCT116 xenografts treated with BCAA restriction, FOLFOX (oxaliplatin 5 mg/kg, 5-FU 50 mg/kg), or their combination for 3 weeks. D Western blot analysis of mTORC1 pathway in HCT116 tumors. ECHS1: Enoyl-CoA hydratase-1, BCAA: Branched-chain amino acids, KRAS: Kirsten rat sarcoma viral oncogene homolog, MRTX1133: the KRAS G12D inhibitor. pERK: Phosphorylated-extracellular signal-regulated Kinase, pAKT: Phosphorylated-Protein Kinase B(PKB), pS6: Phosphorylated-Ribosomal Protein S6, p4EBP1: Phosphorylated-Eukaryotic Translation Initiation Factor 4E-Binding Protein 1. [file 13046_2025_3399_MOESM5_ESM.tif]
